# Not as Bad as I Thought: Consumers' Positive Attitudes Toward Innovative Insect-Based Foods

**DOI:** 10.3389/fnut.2021.631934

**Published:** 2021-06-16

**Authors:** Juliana Rotunno Junges, Natália Rohenkohl do Canto, Marcia Dutra de Barcellos

**Affiliations:** ^1^Department of Administrative Sciences, School of Administration, Federal University of Rio Grande do Sul, Porto Alegre, Brazil; ^2^Department of Management, MAPP Centre for Research on Value Creation in the Food Sector, Aarhus University, Aarhus, Denmark

**Keywords:** consumer attitude, food neophobia, insect-based food, acceptance, innovation, consumer response

## Abstract

Considering that the demand for food will increase by 70% by 2050, consuming insect-based foods appears as a protein alternative due to their nutritional quality and low environmental impact. However, there is a need to investigate the acceptance of these innovative foods, especially in traditional meat-eating markets, such as in Southern Brazil (land of the Gauchos). The purpose of this manuscript is to analyze consumers' attitudes toward innovative insect-based foods. The methodological procedures were divided into two stages. In the qualitative stage, 14 interviews were conducted regarding meat consumption habits. In the quantitative stage, a survey was carried out with 433 consumers. A factor and cluster analysis were performed, and two different groups of consumers were found. The results clearly show a segment with positive attitudes toward insect-based foods. This group had a low degree of neophobia. The products were perceived as not tasty and disgusting, but they were considered modern, with high nutritional value, positive, safe, and beneficial to the environment. Appearance, price, and packaging, combined with flavor, proved to be the attributes considered by consumers as the most important for their acceptance.

## Introduction

The Food and Agriculture Organization of the United Nations (1) estimates that food production will increase by 70% due to the population increase that will occur until 2050. Current cereal production of 2.5 billion tons is expected to grow to 3 billion tons per year, and meat production is expected to increase by more than 200 million tons (1). The increase in food cultivation, especially animal husbandry, will have a negative impact on the environment (2). Thus, alternatives that replace the consumption of animal proteins are emerging and gaining strength in the market, such as insect-based foods (3).

The literature estimates that there are more than 2,100 species of edible insects, most of which are found in tropical countries (4, 5). Besides, they cause less environmental impact since they produce lesser amounts of greenhouse gases and require lesser amounts of land for their production compared to other sources of animal proteins (5). Due to its high reproduction rate, the scale production of insect-based foods would not impact biodiversity (5). Besides, consuming insect-based foods brings several nutritional benefits, such as calcium, vitamins, minerals, fibers, and high levels of proteins and iron (6).

Despite the existing benefits, consumers' attitude is a barrier to introducing insect-based foods in Western societies (7). According to Allport [(8), p. 150], attitudes “are predispositions learned to respond to an object or a class of objects consistently favorable or unfavorable.” This concept has several implications for the study of the consumer's behavior. The first is that attitudes are learned, i.e., they develop through the consumer's direct experience with the product, through the information he obtains with other people, and the individual's exposure to communication vehicles (9). The second is that attitudes are predispositions and therefore reside in the mind. This aspect has a motivational factor involved, since the attitudes “can impel the consumer toward a certain behavior or move him away from a particular behavior” [(9), p. 171]. The third implication is that attitudes are consistent, i.e., they reflect the individual's behavior concerning a particular object (10). Besides, attitudes are affected by situational factors, which means consumers can have different attitudes toward the same object depending on the context. This approach must be taken into account when analyzing the consumer's attitude (9).

Behavior is also influenced by external factors, such as the individual's culture. Schiffman and Kanuk [(9), p. 280] define culture as “the total sum of beliefs, values, and learned customs that direct the consumer behavior of the components of a given society.” It reflects numerous factors, such as regional characteristics, ethnicity, religion, language, and shared fundamental values. Thus, knowing a society's culture helps entrepreneurs predict consumers' acceptance of their products (9).

Van Huis et al. (5) claim that insects are a food taboo in westernized societies since they are perceived as dirty, disgusting, and dangerous. They “are still viewed as pests by a large majority of people, despite the increasing literature pointing to their valuable role in the diets of humans and animals” [(5), p. 141]. Other researchers attribute this rejection to cultural issues inherent to the food purchase process (11). Another barrier is food neophobia—fear of trying innovative foods—which is even more pronounced in animal origin products (12).

It is important to note that consumers' attitudes toward a product or brand are not permanent. Thus, knowing how to conduct strategies to modify attitudes is essential for any marketing professional who wants to insert a new product on the market or acquire more consumers, making them from potential to real ones (9). To change consumers' attitudes, it is first necessary to know people's current attitudes toward the product and then understand which strategy should be used.

Given this scenario, the inclusion and commercialization of insect-based foods in Brazilian society emerge as a sustainable protein alternative, which can reduce the environmental impacts of food production. However, before introducing the product to the market, it is necessary to ascertain consumer acceptance. Furthermore, there is very scarce research concerning the consumer acceptance of edible insects in Latin America (13, 14). Thus, this research aims to analyze consumers' attitudes toward innovative insect-based foods. The Brazilian state Rio Grande do Sul was chosen as the focus of this research due to the region's high meat consumption.

In addition to this introduction, this article presents a brief literature review on the consumption of insect-based foods (section The Introduction of Insect-Based Foods in Western Countries), the methodological procedures adopted (section Methodological Procedures), results (section Results), and final considerations (section Discussion).

## The Introduction of Insect-Based Foods in Western Countries

The food sector is usually described as being a segment of the market with slow growth, considering that little is invested in research and development to introduce innovations in it, being, therefore, very conservative (15). Innovation in the food sector is also more complex than thought at first, as it involves all actors in the production chain that must be carefully coordinated to achieve the desired results and requires the approval of regulatory bodies (16). Besides, companies have to deal with consumer acceptance and their attitude toward new products and modes of production, a crucial factor for food products to be successful in the market (17). Despite this, with the increasing level of competitiveness, innovation is no longer an option for organizations, becoming a necessity (15) for those who want to please contemporary consumers who increasingly demand unique flavors and they are concerned with having healthy meals that meet their ideals and values (16).

The consumption of insect-based foods fits in this context because this new way of eating is an innovation for Westernized societies. However, it has been suffering resistance in countries that have introduced these new products, such as hamburgers based on cricket, ant flour, chips, and processed cereal bars. Researchers justify this aversion to insects in several ways, however, it is considered a curious historical fact, since the consumption of insects, in its natural form, is not a recent practice, it has been part of the human diet for millennia, having its first findings described in the Bible (5). Despite this, this eating habit has not expanded worldwide, being more adhered to today by Eastern societies. Thus, it is believed that it was because of the little use of these animals (except for bees and silkworms) that they were not successful on a large scale. Besides, its seasonality may have contributed to the loss of interest in human consumption (7).

Van Huis et al. (5) consider that negative attitudes toward insects may have started with the advent of agriculture when they started to be seen as pests. Berenbaum (18) suggests that the human body is biologically prepared to be afraid of these animals, as a defense mechanism, given that spider, wasp, scorpion, and bee stings, even the not deadly ones, are unpleasant for humans. Thus, this would explain why they are seen with fear, disgust, and dislike, and are also considered by many as a primitive and poor way of eating (19).

In addition to biological, rational factors, and human emotions, culture also contributes to these foods being rejected, as it determines what is edible and what is not (20). Since childhood, people learn how to eat, discerning which foods are healthy and good for the body, assigning, over time, different meanings, associations, and classifications to foods. Thus, due to each society's food culture, people choose what they already know, avoiding the unknown (21).

In Latin America, entomophagy has been practiced by indigenous people for a long time and insects are seen in some areas as a delicacy. Hence, it would be logical to think that consumer acceptance toward edible insects is generally higher in Latin America. Nevertheless, a big part of the urbanized population in the region despises their consumption and associates it with poverty and “Indianness” due to ethnocentric reasons. Studies are scarce (13) and the few examples available come from Brazil, where Cheung and Moraes (22) reported that most consumers associate insect consumption with the words “disgust” and “no.” Lucchese-Cheung et al. (23) found a very low intention to consume insects as food and respondents had not been exposed to consuming insect-related food products. They would rather accept food products with insects disguised as an ingredient.

In this regard, another point to be considered concerning the rejection of insect-based foods is food neophobia. Pliner and Salvy (24) conceptualize neophobia as resistance to eating new foods, avoiding them, and prioritizing family foods. Rozin (25) and Pliner and Salvy (24) present three factors that lead to food rejection: (i) not liking the sensory characteristics, (ii) fear of negative consequences when eating the product, (iii) disgust at the origin and nature of the food.

These obstacles hinder the acceptance of insects as input for food production and end up favoring the mass production of other types of proteins. Thus, due to their variety and dispersion around the world, the use of insects for food production fits in a context where the population and the demand for food is growing, and solutions with low environmental impact are needed. Therefore, they are both an economically and environmentally more sustainable option for those entrepreneurs who wish to invest in this niche market. Nevertheless, food insecurity and pressures for companies to adopt sustainable production practices are also important factors when it is suggested that insects be included in sustainable diets and may be the leading food supplements suggested due to their nutritional value (5).

## Methodological Procedures

An approach with mixed methods was adopted to achieve the proposed objective. This approach is characterized by the collection and combination of qualitative and quantitative data in a single study, comparing the information obtained (26).

**Figure 1:**
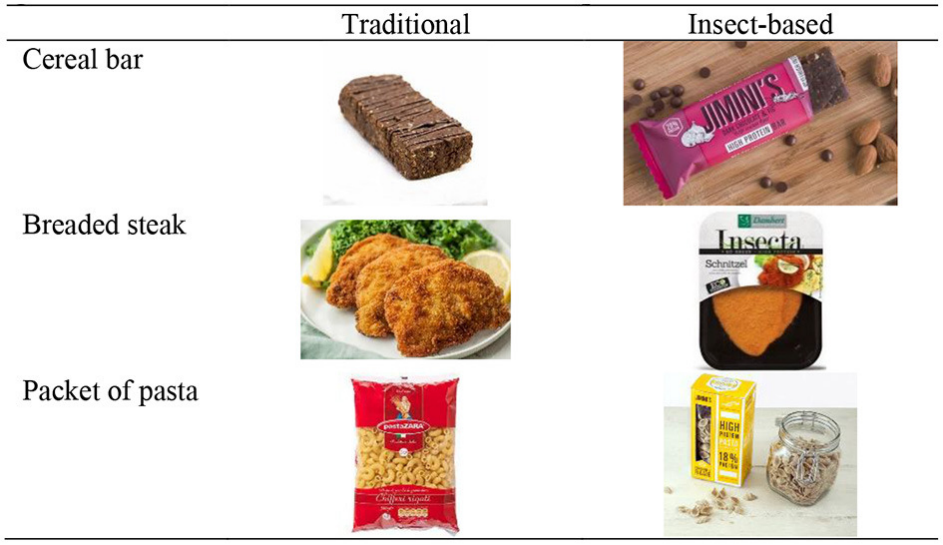
Photos of traditional and insect-based products shown to consumers. Cerel bar traditional: Gluten Free on a Schoestring. Homemade protein bars (2017). Available online at: https://glutenfreeonashoestring.com/homemade-protein-bars/ (accessed May 1st, 2019); Cereal bar insect-based: The Guardian. Grubs up: Carrefour offers Spanish shoppers insect-based snacks (2018). Available online at: https://www.theguardian.com/world/2018/apr/19/grubs-up-supermarket-offers-spanish-shoppers-insect-based-foods (accessed May 1, 2019): Breaded steak traditional: The Pruce Eats, Authentic Wiener Schnitzel Recipe. Available online at: https://www.thespruceeats.com/wiener-schnitzel-recipe-1447089 (accessed May 1, 2019). Breaded steak insect-based: Pinterest. Nuggets, burguer et escalope d'insectes par Damhert (Belgique). Available online at: https://br.pinterest.com/pin/324892560598792309/visualsearch/?x=16&y=10&w=517&h=320 (accessed May 1, 2019). Pasta traditional: SXM Distributors. Pasta Zara Garmignone. Available online at: http://sxmdistributors.com/dt_gallery/pasta-zara/pasta-zara-garmignone-500g-550x550/ (accessed May 1, 2019). Pasta insect-based: Twitter. Jimini's protein bar (2017). Available online at: https://twitter.com/jiminisfood/status/905116066610851840 (accessed May 1, 2019).

For the qualitative stage, after a literature review on the subject, interviews were conducted with a semi-structured script. The questions investigated food consumption habits, protein consumption, and attitudes toward insect-based foods. Photos of insect-based products were also presented (Figure 1). The selection criterion of the interviewees was that they had different meat consumption habits since it is the most targeted protein source due to environmental and health impacts (27). The interviews were conducted in April 2019 and lasted between 25 and 40 min. They were recorded with the interviewees' consent and later transcribed. The data were categorized in the Excel software in order to facilitate the visualization of the responses and to identify similarities and differences in the attitudes of the interviewees.

After the interviews, respondents were classified into four groups according to their meat consumption: (1) Daily consumption—they consume some type of meat daily and do not want to reduce consumption, (2) Daily consumption, reduction intention—consume some type of meat daily, but want to reduce consumption, (3) Reduced consumption—consume meat a maximum of three times a week, (4) No consumption—do not consume animal proteins, but do consume dairy products and eggs. The interviews were conducted until the answers were saturated (28). In all, 14 consumers from the Rio Grande do Sul state, Brazil, residents in Porto Alegre and the Metropolitan Region, were interviewed (Table 1).

**Table 1:** Profile of respondents in the qualitative stage.

**Group**		**Interviewee**	**Gender**	**Age**	**Occupation**
1	Daily consumption	A	Male	20	Student
		B	Female	26	Accountant
2	Daily consumption, reduction intention	C	Male	23	Manager
		D	Male	23	Accountant
		E	Male	25	Engineer
		F	Female	48	Assistant
		G	Female	54	Doctor
		H	Male	61	Doctor
3	Reduced consumption	I	Female	83	Housewife
		J	Male	87	Retired
4	No consumption	K	Female	22	Student
		L	Female	23	Nurse
		M	Female	25	Lawyer
		N	Female	60	Saleswoman

For the quantitative stage, an online survey was applied. The questionnaire was prepared based on previous studies with 37 questions, organized in six sections.

The first section consists of a filter question about the respondent's residence since the research is aimed at consumers in the state. The second section identifies the consumption habits of white and red meat and asks about the interest in reducing this consumption. For respondents who do not consume meat, the motivations for doing so were questioned. For respondents wishing to reduce meat consumption, the type of animal protein targeted and the motivations were questioned.

In the third section, the Pliner and Hobden (29) food neophobia scale was used, composed of 10 statements measured on a 7-point Likert scale—where 1 means “totally disagree,” 7 “totally agree,” and four represents neutrality.

In the fourth section, it was asked whether the participant had any knowledge about insect-based foods. From this, three pairs of photos were presented (Figure 1). Each pair consisted of a traditional food and the same food partly made up of insects. Three popular and familiar products were chosen: cereal bar, breaded steak, and a packet of pasta. Since the consumer acceptance of insect-based products is very low amongst westerners, introducing them in a non-visible form is more advantageous. The acceptance of an insect-based product is also likely to increase with the increasing familiarity of the product [such as bread, biscuits, or pasta that include insect flour, according to Kulma et al. (30) and Rumpold and Langen (31)].

In the fifth section, Hartmann and Siegrist's (32) semantic differential scale of 10 points was used, identifying the respondents' attitudes by the phrase “I think insect-based foods are ___.” This scale is composed of two adjectives at each extreme, representing opposite poles (33). The adjectives pairs used were: primitive/modern, low nutritional value/high nutritional value, not tasty/tasty, negative/positive, unsafe/safe, harmful to the environment/beneficial to the environment, disgusting/pleasant. Afterward, it was questioned which attributes would be important in insect-based products, and there was also the option to indicate that there was no interest in consumption. The next question asked about the desire to consume insect-based foods in the future. The sixth section was about the socio-demographic profile of the respondents.

Before collection, a pre-test was carried out with four consumers to improve the understanding and clarity of the questions. After correcting the survey instrument, the questionnaire was sent by e-mail and shared on social networks. The collection was carried out between May 10 and 18, 2019, obtaining 443 responses. The 33 respondents who were not gauchos were removed, leaving 410 valid responses.

The information was exported to Excel, and SPSS software was used for data analysis. A factor and cluster analysis were also carried out in order to segment consumers according to their attitudes toward products. Factor analysis and cluster analysis are distinct, but complementary. They provide powerful multivariate statistical tools for the exploration of similarity relationships among subjects and/or variables. Factor analysis has the ability to reduce an unwieldy set of variables to a much smaller set of factors, suitable for simplifying complex models. Cluster analysis, on the other hand, is used to address heterogeneity in each set of data. It is a form of categorization, suitable for classifying objects (subjects, persons) according to certain criteria (34).

## Results

This section presents the research results. Section Qualitative Stage analyzes qualitative interviews. Section Quantitative Stage analyzes the survey results.

### Qualitative Stage

The results of the interviews are divided into four sections. Section Food and Protein Consumption Habits presents the profile of the interviewees regarding their consumption habits. Section Perceptions of Insect-Based Products explores initial insights into insect products. Section The Behavior Toward Insect-Based Foods, Considering Meat Consumption presents an analysis of the attitude of consumers by the groups in which they were segmented. Section Market Aspects deals with perceptions in relation to market aspects (appearance of the product, price, packaging, variety, accessibility).

#### Food and Protein Consumption Habits

Regarding food consumption in general, some of the consumers interviewed are very concerned with the amount and quality of food, checking labels, and packaging, while others are less involved with these issues. Women expressed greater concern about the environmental impacts of food production. About the consumption of proteins, some interviewees did not know which foods have this nutrient. Respondents who do not consume any type of meat demonstrated greater knowledge on the subject, reporting various sources of substitute foods. The type of meat that the interviewees mentioned seeking to reduce or eliminate from their consumption is beef. The main reasons cited in this regard were: the longer digestion of meat by the body, which causes a feeling of discomfort and indisposition, and the high levels of fat in this protein.

#### Perceptions of Insect-Based Products

The initial perceptions about insect-based foods were astonishment, shock, strangeness, and disgust. Respondents were unaware of the marketing of these products and never had access to them. Interviewee C believed that eating insect-based foods and entomophagy were the same subject: “I imagine an insect will come on my plate, a mosquito on my pasta.” Other respondents referred to this form of eating as primitive times. There was also a concern on which insects would be used, for example, whether cockroaches would be included: “I think that depending on the insect it is disgusting, ants are not, but the other animals are disgusting” (Interviewee L). Another question was related to the form of production since it is popularly known that insects can spread diseases, thus, the certainty that the food is safe was considered a condition. Only respondents E and H knew that insects have high nutritional quality.

After showing photos of insect-based products that are sold abroad (Figure 1) and providing some information about the nutritional benefits of these foods and their production process, some consumers became curious about them. Thus, it was found that curiosity was the biggest motivation to try the products, especially in relation to taste. Consumers seeking to reduce meat consumption are also motivated by nutritional quality, especially in relation to proteins. This aspect is attractive for those focused on health and who seek to control the amount of protein ingested. Only Interviewees B and D cited motivations related to environmental issues.

#### The Behavior Toward Insect-Based Foods, Considering Meat Consumption

This section analyzes the attitude of consumers according to the meat consumption group in which they were classified in Table 1. In the end, the difference in attitude between the groups is exposed.

Consumers in the daily meat consumption group were more cautious and suspicious about the products but did not reject them. These consumers stated that they are more traditional in their diet, eating the same products daily, which may have contributed to their hesitation:

It's strange, a peculiar thing, maybe because it's not in my daily consumption, because I don't have an affinity for this type of product. I think that maybe flour or something that I knew would be powdered, I would eat, but the rest I don't know. Perhaps I could adapt if I were better informed (Interviewee A).

Consumers in the group that eats meat daily, but intends to reduce consumption, showed a different attitude toward products. Interviewees C, D, E, and G showed a lot of interest in consuming insect-based foods. Participant D stated: “I think, for sure, if there was more investment in this, it would have already been disseminated in people's culture.” Participants D, C, and G reacted with surprise when they learned of the benefits that the products could bring to their health, which implied reflections on why these products are not yet offered in the Brazilian market: “it will surely be the food of the future” (Interviewee D). Interviewee G was the only participant over 45 years old who had a positive attitude in this group. Concerned about food, she believes that insect-based products can bring new options to the menu, as long as they are easy to prepare.

Participants F and H did not show much interest and motivation to purchase insect-based products and, therefore, did not have a favorable attitude. They proved to be more conservative about their diet, seeking to acquire foods they already know and are used to, since there is a great concern with the effects on the body that new combinations of ingredients can cause.

Consumers in the group that has already reduced consumption of meat have shown to be conservative, as they do not like to try new dishes in general. They had a negative attitude toward the products, stating that they would only eat in cases of extreme necessity, such as the lack of options in the market, or if their survival relied on it:

if it was necessary, last case scenario, I would eat it, only if there we lacked the food that we are used to [.] Why use this product here if it is not necessary yet? I would not recommend it to anyone if I had the chance to eat more common stuff. Better to take a fish out of the sea than to grab an insect out there that we don't know where it has been (Interviewee I).

These consumers would only consume insect-based foods if they were very healthy or had an exceptional taste.

Finally, in the group of consumers that does not consume meat, respondents K, L, M demonstrated a negative attitude toward insect-based foods, with their values and principles as the main argument:

I might be curious to taste it, but as it goes against my principles, I would not eat. I would not feel comfortable, I would eat guiltily because I think they are living beings, they are important for the ecosystem, it is not something that draws me (Interviewee K).

The three interviewees justify their lack of meat consumption due to concern for animal welfare, slaughter experience and testimony, the influence of other people, and the belief that meat is food without energy and without life: “I don't eat death, I eat life. I eat plants that are lives” (Interviewee M). All argued that, even if they were curious to taste insect products, they believe that the total elimination of meat is a more viable and sustainable alternative than the production of insects: “Maybe I would feel curiosity, but it would not be something strong enough for me to try it” (Interviewee K). However, despite faithfully defending vegetarianism (eliminating animal proteins in food) and veganism (applying this philosophy to all aspects of an individual's life), Interviewee O stated:

I think meat is more disgusting than insects because it's the meat of the animal there. I think it is an animal that does not need to be consumed. In insect products, you don't see the animal, it's powdered, so you don't see it, you don't really know. It's a less disgusting thing, but it's weird to think that we're consuming insects.

Unlike the other respondents, whose motivation is related to the animals' lives, Interviewee N eliminated meat from her diet because she did not like the taste. She showed a positive attitude toward insect-based foods, which, in her opinion, would be a great alternative as an iron source, she was also interested in the amount of protein in these products. This shows that not all consumers who do not eat meat have the same attitude, therefore, it is necessary to investigate and explore the causes of the change in eating habits. In the sample of this research, this motivation was what distinguished the acceptance or rejection of the products.

Comparing the four meat consumption groups, consumers who eat meat daily showed a more neutral and hesitant attitude toward products. Consumers who eat meat daily but intend to reduce showed more enthusiasm and willingness to consume the products. However, two participants in this group showed an attitude of rejection. In this group, participants with a positive attitude like to try new foods and are more concerned with maintaining a healthy diet, those who had a negative attitude appear to be more neophobic and had a more sedentary lifestyle. Consumers who had already reduced the consumption of meat showed a negative attitude, similar to those who rejected products in the previous group. Among the consumers who do not eat meat, the ones who rejected the insect-based products claimed that they do not eat meat because they are concerned with animal welfare. The interviewee with a positive attitude does not eat meat because she does not like the taste. Thus, the results indicate that the reasons why consumers change their eating habits (concern for animal well-being and their own health) and also food neophobia must be taken into account when analyzing the attitude toward products based on insects.

#### Market Aspects

When the interviewees were asked about the attributes of the products that they consider important for acceptance in the market, the flavor was the most mentioned aspect, requiring that the foods have a “neutral to good” taste. Regarding the appearance and packaging of the product, women were the ones who most emphasized its importance, for men, the price gained prominence. Interviewee N's account demonstrates the importance of presenting the product to her:

I think the spices are important and it has to have a beautiful color, a good smell. Something that manages to attract through our sense of smell that you think and look at that product, “wow, look, how beautiful.” Our senses have to be drawn by the product.

It was also reported the need for the ingredients to be well-powdered and processed, having shapes and textures of familiar products, thus avoiding the appearance of an insect. In this sense, for respondent L, not showing the photo of the insect can be a strategy to be adopted by companies. However, the indication of which insects are used and the breeding process must be displayed on the packaging to bring confidence and security in the food. In addition, the interviewees mentioned that the packaging must be “pleasant, attractive, beautiful,” produced with sustainable material and with information that makes consumers aware of the product's benefits for the environment. Although they reported the importance of sustainable packaging, only two consumers reported this factor as a motivation for purchase.

Regarding the price, the interviewees affirm that it must be competitive in relation to other products:

I think [consumers] would even pay more for being exotic, but if it is to enter the market as an alternative source of protein, to enter day-to-day life, it cannot be expensive. People will not stop eating meat that is much more palatable and is already our daily life, to eat insects that would not be something so delicious. Especially because meat is in our culture, so the person would not want to eat insects (Interviewee K).

Consumers also justified that, due to unusual ingredients and the need to make it an alternative to other products, insect-based food should be economically attractive: “Nothing will do us any good to replace the food we have today if we don't it is economically sustainable and the taste is bad, there has to be a balance in everything” (Interviewee C). Interviewee H said that if the products are too expensive, he would not buy them often, and also conditioned their purchase on health benefits.

Interviewee G would also pay more due to the benefits and nutritional quality of the product, compared to organic products, which she buys despite its high price. She also addressed the importance of products being available in different compositions, having different flavors and textures: “I think that products have to have different presentations, like cream, flour, liquid.” In addition, he reported the need to have easy access to products, having to be available in large markets, since, due to his daily life, he would not be able to go to specific stores to look for the products. Only consumer C stated that he would go to small stores looking for the products if they were not available in large markets.

These are the main insights generated from the qualitative stage of the work. In the next section, the results of the quantitative phase are presented, which allowed verifying the predominance of these initial impressions in a larger sample.

### Quantitative Stage

This section presents the survey results. Section Respondents' Profile presents the profile of the participants and their habits regarding meat consumption. Section Attitude of Participants Toward Insect-Based Foods investigates the participants' knowledge of insect products and their attitude toward them. In section Cluster Analysis, the market is segmented according to the attitude of the participants, and, finally, in section Market Implications, marketing strategies are suggested for companies that want to invest in these foods.

#### Respondents' Profile

Table 2 summarizes the demographic characteristics of the 410 survey respondents. The predominant profile is women up to 29 years of age, with postgraduate degrees and monthly family income between 3 and 6 minimum wages.

**Table 2:** Demographic characteristics of the respondents.

	***n***	**Percentage (%)**
**Gender**		
Male	147	36
Female	263	64
**Age**		
Up to 29 years	113	28
Between 30 and 39 years	66	16
Between 40 and 49 years	58	14
Between 50 and 59 years	83	20
More than 60 years	90	22
**Level of education**		
Incomplete higher education	153	37
Complete higher education	105	26
Graduate degree	152	37
**Family income**		
Up to R$ 2,994.00	68	17
Between R$ 2,994.01 and R$ 5,988.00	112	27
Between R$ 5,988.01 and R $ 9,980.00	81	20
Between R$ 9,980.01 and R $14,970.00	52	13
Above R$ 14,970.01	97	24

Table 3 presents descriptive statistics of questions related to meat consumption habits and intention to reduce meat consumption. The great majority of respondents eat meat on a daily basis. Those who consume meat were asked whether they intend to reduce the amount eaten or to stop eating any category of that protein. Thus, 38% (146 consumers) answered in the affirmative. Although these data do not represent the majority, there is a great tendency to decrease this consumption, as was also seen in the exploratory stage.

**Table 3:** Descriptive statistics of meat consumption habits and intention to reduce meat consumption.

	**n**	**Percentage (%)**
**Meat consumption habits**		
Daily consumption of meat	269	66
Meat consumption to 3–4 days a week	72	18
Meat consumption to 1–2 days a week	45	11
Do not consume meat, but consume dairy products and eggs	17	4
No consumption of animal foods	5	1
**Types of meat that respondents intend to reduce or stop**		
**consumption^a^**		
Beef/Bovine meat	131	45
Pork	43	15
Poultry/Chicken	39	13
Lamb meat	33	11
Fish	22	8
Other types of seafood	21	7
**Motivations for reducing or not consuming meat ^*b*^**		
Improving my quality of life in general	102	29
Concern with animal welfare	91	26
Improvement in health	82	24
Environmental concern	49	14

a *Applicable to respondents that consume meat*.

b *Applicable to respondents that wish to reduce meat consumption or who do not consume*.

Beef/bovine meat ranked first among the types of meat that respondents want to reduce or eliminate from their consumption, confirming the results of the previous stage. Then came pork and poultry/chicken. Those who wish to reduce meat consumption or who do not consume were asked about their motivations. The item “improving my quality of life in general” was the most cited as a motivator, followed by the concern with animal welfare and a prospect of improvement in health were the most marked. Few mentioned the environmental concern, of which 84% are women.

#### Attitude of Participants Toward Insect-Based Foods

Initially, the respondents' knowledge about insect-based foods was verified after presenting the following concept: “they are foods with a traditional format in which insects are part of the product's composition. These insects are bred in captivity, in a regulated and wholesome way, so that they are processed and crushed, becoming food.” Forty-five percent of respondents said they knew the practice of eating insects only as part of the eating habits of some people and countries, 38% said they did not know about the subject, and 18% expressed some knowledge. Thus, the sample is characterized as poorly informed about processed foods that have insect-derived protein as part of their ingredients.

**Figure 2:**
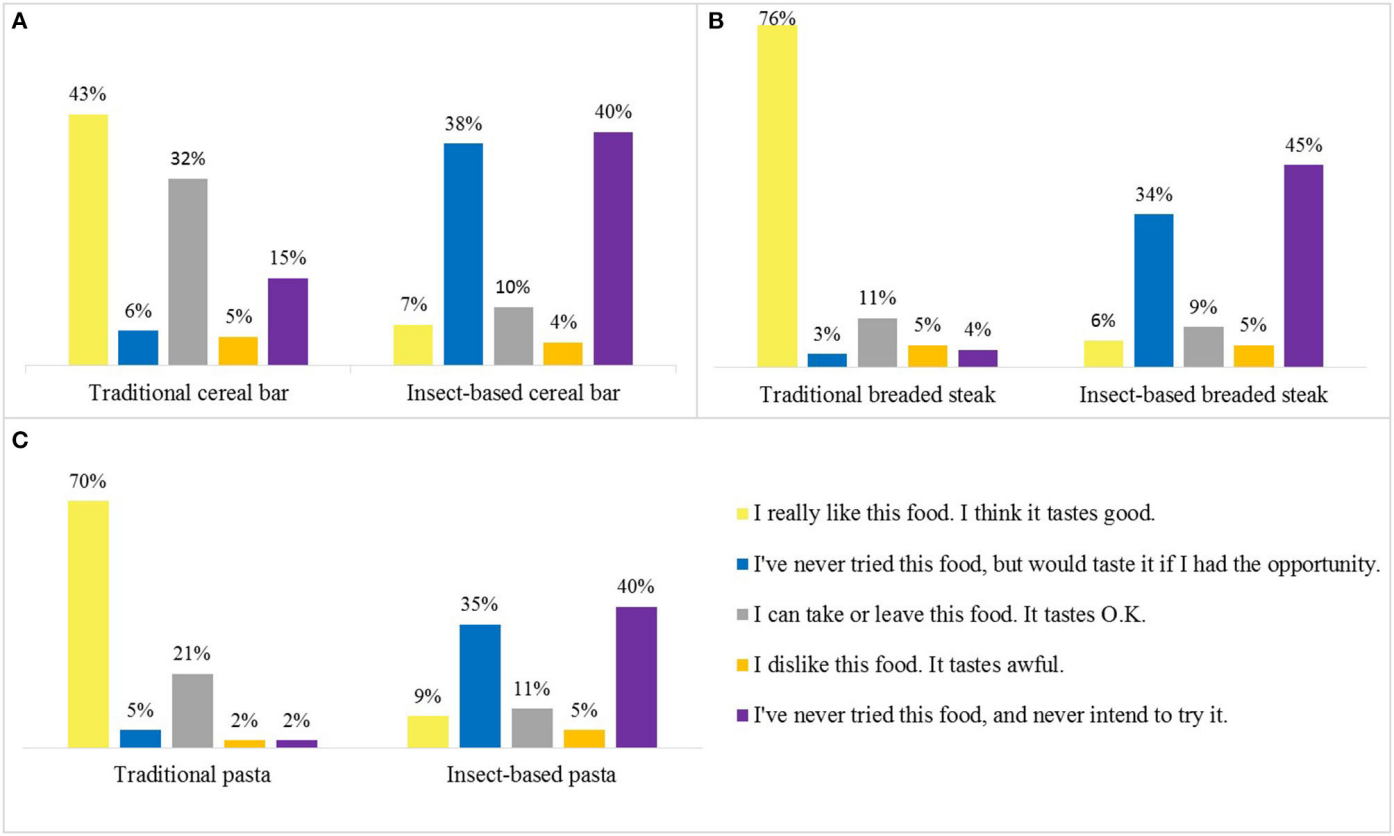
Consumers' attitudes toward traditional and insect-based cereal bar **(A)**, breaded steak **(B)**, and pasta **(C)**.

Subsequently, three pairs of photos were exposed to the participants. The first photo of each pair represented a traditional food product, and the second the same type of food but made in part with insect flour. The products chosen were a cereal bar (snack), breaded steak (protein), and pasta (carbohydrate). For the analysis, the favorable responses to the products (represented by the colors yellow and blue in Figure 2) were grouped with the unfavorable opinions (represented by the colors orange and purple in Figure 2).

Regarding the cereal bar, the traditional version was well-accepted, but also, a large fraction of respondents showed impartiality. In the insect-based version, 45% of consumers showed a positive attitude, 44% rejected the product, and 10% remained neutral. Thus, the attitude of consumers was divided into two poles, on the one hand, a group that would strongly like to try the product, and, on the other, with strong aversion. Thus, a positive attitude was found in relation to the two versions of the cereal bar, however, the insect-based product had a greater rejection.

Regarding breaded steak, in the traditional version, 79% of respondents exposed a positive attitude about the product, 11% are neutral, and 9% rejected. Therefore, this product has high acceptance. In the insect-based version, 40% of the sample showed interest in the product, 50% had a negative attitude, and the others had a neutral opinion. Once again, two consumer profiles were found that are divided in their opinions, however, the rejection was greater in relation to the cereal bar.

Regarding pasta, in the traditional version, 75% of respondents say they like the food, 4% say they don't like it, and 21% remain impartial. In the insect-based version, there was, again, a division in the attitude of consumers: 44% expressed a positive attitude, 45% did not like the product, and 11% were neutral. The grouped percentages obtained were similar to those of the cereal bar.

In short, among traditional products, the breaded steak was the most accepted by consumers, followed by pasta and cereal bars. However, this relationship was not the same for insect-based products, with the cereal bar being the most accepted product, followed by pasta and breaded steak. Therefore, the results indicate the cereal bar as the product that may have greater acceptance in the market, while breaded steak is likely to have the least acceptance.

After the photos, the attitudes of the participants toward seven attributes were verified, as shown in Table 4. Values close to 10 indicate greater agreement with the attribute on the right, values close to 1 indicate greater agreement with the attribute on the left. Thus, in general, it was found that insect-based foods were predominantly considered modern, of high nutritional value, not tasty, positive, safe, beneficial to the environment, and disgusting. However, the proximity of some values to the central point (6) indicates that consumers do not yet have a clear position on some characteristics investigated.

**Table 4:** Perception regarding insect-based foods.

**I think insect-based foods are:**	**Average^a^**	***SD***
Primitive/Modern	5.70	3.25
Low nutritional value/High nutritional value	6.72	2.93
Not tasty/Tasty	4.12	2.62
Negatives/Positives	5.41	2.99
Unsafe/Safe	5.22	2.90
Harmful to the environment/Beneficial to the environment	6.09	2.98
Disgusting/Pleasant	3.77	2.74

a *Values close to 10 indicate greater agreement with the attribute on the right; values close to 1 indicate greater agreement with the attribute on the left*.

#### Cluster Analysis

In order to segment consumers according to their attitudes toward insect-based foods, the base and segmentation variables were first defined. As a basis, the semantic differential scale was used, which represents the attitude of consumers toward insect-based products. As variables to characterize the formed segments, the Pliner and Hobden (29) food neophobia scale and the question about the willingness to taste insect-based foods were used.

Afterward, factor analysis was carried out to identify factors that synthesized the ten statements of the neophobia scale. The Kaiser-Meyer-Olkin index (KMO) of 0.84 was obtained, indicating that the factorial analysis performed was valid and acceptable, with a strong correlation between the crossed variables. The variables were grouped into two factors (Table 5): the first factor included variables related to the consumption of new foods and of different ethnicities, in the second factor, there were the variables related to distrust and demand in relation to innovative foods. From these factors, two variables were created, called “neophobia in relation to new foods and of different ethnicities” (Factor 1) and “distrust and demand in relation to new foods” (Factor 2).

**Table 5:** Factor analysis of the Pliner and Hobden food neophobia scale (29).

**Standard matrix**	**Factors**	
	**1**	**2**
	**Neophobia in relation to new foods and of different ethnicities**	**Distrust and demand in relation to new foods**
I am constantly trying new and different foods. [reversed]	0.760	
I don't trust new foods.		
If I don't know what a food contains, I don't try it.		0.762
I like foods from different countries. [reversed]	0.817	
Food from other countries seems too strange to be consumed.		
At social events, I try new foods. [reversed]	0.789	
I am afraid to eat foods that I have never tried before.		0.610
I am very picky about the foods I choose to eat.		0.763
I eat just about everything. [reversed]		
I like to go to new restaurants with food from other countries. [reversed]	0.872	

Market segmentation was performed through a cluster analysis using the SPSS software. The “Two-Step Clusters” algorithm was used, seven inputs were used for the segmentation that represents the consumers' attitude toward insect-based foods.

Two segments were obtained, with an average quality of differentiation (Table 6). The first cluster has 184 respondents (44.9%) and a more favorable attitude toward insect-based products. The second cluster has 226 consumers (55.1%) and a less favorable attitude toward products. Therefore, the first cluster of “consumers with a favorable attitude toward insect-based foods” was named, and the second cluster of “consumers with an unfavorable attitude toward insect-based foods.”

**Table 6:** Segmentation according to the attitude toward insect-based products.

		**Cluster 1 averages**	**Cluster 2 averages**
		**(*n* = 184)**	**(*n* = 226)**
Base of segmentation	Negatives × Positives	7.83	3.45
	Unsafe × Safe	7.49	3.38
	Disgusting × Pleasant	5.85	2.07
	Not tasty × Tasty	5.80	2.75
	Harmful to the environment × Beneficial to the environment	7.88	4.62
	Primitive × Modern	7.62	4.14
	Low nutritional value × High nutritional value	8.24	5.42

Regarding the rate of food neophobia (Table 7), the first cluster did not demonstrate neophobia in relation to consuming innovative foods and was also undemanding and suspicious about these products, the second cluster showed a degree of positive food neophobia for both factors. In other words, the greater the food neophobia of consumers, the lower their attitude toward insect-based foods. With regard to the desire to consume the products in the future, the result was consistent, since Cluster 1 showed a positive attitude and Cluster 2, negative.

**Table 7:** Food neophobia and willingness to consume insect-based products in the future.

	**Cluster 1**	**Cluster 2**
Neophobia in relation to new foods and of different ethnicities	−0.32	0.26
Mistrust and demand for food	−0.26	0.21
Willingness to consume insect-based products in the future	Yes (69.21%)	No (83.18%)

Therefore, there are two segments with similar proportions. This shows that, just as there is an audience that does not want to consume products based on insects (55.1%), there are, on the other hand, consumers who are strongly interested in consuming the products (44.9%). There are, thereby, potential consumers and, as a consequence, an opportunity for entrepreneurs who wish to invest in this niche market.

#### Market Implications

Regarding the most important marketing attributes (Table 8) for consumers to buy the products, appearance was the most important factor (46%). Thus, the more similar insect-based foods are to known foods in terms of shape, color, texture, smell, and taste, the greater the chance that consumers will want to taste them. This familiarity can also contribute to reducing food neophobia, as, as Rozin (25) and Pliner and Salvy (24) claim, one of the factors that lead to the fear of trying new foods is the rejection of sensory characteristics.

**Table 8:** Marketing attributes for consumers buy the products.

Appearance of the product	46%
Price	40%
Packaging	24%
Variety	19%
Accessibility	17%

The price was also mentioned as a very important aspect at the time of purchase (40%). Bearing in mind that the products could enter the market as an alternative to other food sources, contributing to the reduction of environmental impacts, the price will be a crucial element for the products to be accepted by the gauchos. Therefore, it should be initially attractive and competitive in relation to traditional products since it is a product that has a cultural barrier involved. For products to be valued and consumers to pay more for them, there must be other attractive attributes—such as a different flavor—or be easily incorporated into other dishes (35).

As for packaging, 24% of respondents consider this factor to be important. Therefore, in addition to having to be attractive and beautiful, it must contain information about: how insects are created, how food is produced, hygienic-sanitary issues, and the type of insect the product is made of. These issues were also addressed in the qualitative stage, which further emphasizes the importance of the public having access to this information. It is suggested to use the product's origin seal, access to traceability through a QRcode, and obtain certifications as strategies to provide more confidence to the consumer that the product is safe. Finally, the need for the products to be available in different varieties (19%), both in terms of taste, as well as the format and the ease of finding the products (17%), were the two factors considered to be less important.

## Discussion

This research analyzed the attitude of consumers in Rio Grande do Sul toward insect-based foods, providing insights for entrepreneurs who wish to invest in these products. Most consumers consulted consume some type of meat every day, however, there is already a percentage of consumers interested in reducing the consumption of this protein (38%). Cattle meat was the most indicated as a target for reducing or eliminating diets both in the qualitative and quantitative phase. The main motivators cited to reduce meat consumption were improvements in quality of life, concern for animal welfare, and improvements in health. In both stages of the research, consumers had little knowledge about insect-based products, however, most of them already knew about entomophagy.

In the qualitative stage, consumers' initial perceptions about food were explored. Many interviewees at first believed that the consumption of insect-based products and the consumption of insects in their natural form were the same issue. There was a concern with the type of insect used and in relation to hygienic-sanitary issues since people see them as transmitters of diseases. Curiosity, especially in terms of flavor, was the most mentioned motivation to taste the products.

Regarding the profile of consumers more willing to taste the products, in the qualitative phase, it was observed that they are those who like to taste different foods, new combinations of recipes and are also more concerned with the health benefits of food. Consumers who fit this profile were those who seek to reduce meat consumption or who do not eat meat because they do not like the taste. In the quantitative phase, this result was confirmed since the segment most interested in the products showed a low suspicion in relation to new foods. Therefore, this consumer profile must be the target audience when products are placed on the market.

Consumers in the qualitative phase who showed a negative or neutral attitude toward products were those who were more conservative in terms of food, seeking to purchase products that they are already used to. Some interviewees imposed conditions to consume the products, such as (i) if it was possible to notice the benefits of the products in the body, (ii) if it had an exceptional taste. Consumers who fit this profile were those who consume meat daily, who seek to reduce meat consumption, and who do not consume because they are concerned with animal welfare. In the analysis of clusters, the segment of consumers less interested in the products showed to be neophobic and very suspicious about new foods. Thus, it can be said that the more neophobic the consumer, the lesser his tendency to want to try insect-based products.

When analyzing the attitudes of the participants in relation to the products—cereal bar, breaded steak, and pasta made from insects—the cereal bar was the product with the highest acceptance and the breaded steak with the highest rejection. This demonstrates a resistance on the part of Gaucho consumers to substitute traditional meat for another source of food. Thus, it is suggested that entrepreneurs who wish to invest in this niche market invest primarily in products such as snacks. The successful introduction of this type of product on the market may contribute to consumers accepting other varieties. It is concluded that insect-based foods are considered modern, of high nutritional value, not tasty, positive, safe, beneficial to the environment, and disgusting by the public in the state.

As a practical implication of the work, it is suggested to entrepreneurs who want to invest in this market that they focus on a restricted number of arguments, however strong, such as the nutritional quality of the products, in order to attract that public that seeks a healthy lifestyle. However, there was also a need to inform the public about the other benefits that these products can bring, such as reducing impacts on the environment compared to other food sources. The need to inform consumers about the benefits of food was also perceived by Lensvelt and Steenbekkers (36) and Lammers et al. (37), who studied the attitude of consumers in Australia and the Netherlands and Germany, respectively. In addition, Lensvelt and Steenbekkers (36) propose that, in order to increase people's acceptance, it is necessary to give them the opportunity to taste insect-based foods, since, in their research, consumers who had previously eaten the products demonstrated a more positive attitude than those who have never tried. Thus, providing samples of products in supermarkets can be a strategy to be used. Additionally, the data obtained indicate that the products should look good, the price will have to be similar in relation to traditional products, the packaging must not only be attractive but must also inform about food production, the type of insect used, hygienic-sanitary issues, and the product must be widely available in large supermarkets. However, none of this will suffice if the products taste bad.

Thus, it is concluded that, although the products are little known by consumers, there is potential to develop this market if an investment is made with the proper positioning, informing consumers about the benefits of the products. However, for that, it is necessary to first regulate the consumption of insects in the country, as was done in Europe recently, guaranteeing food security. It is also important that government entities help to educate these consumers, bringing more information about the way food is produced, showing the importance of consuming insect-based products, just as the FAO has done.

Therefore, this research can provide support to government entities, entrepreneurs, and consumers who wish to promote the consumption of insect-based products and reduce the environmental impacts caused by the current production system. In this context, this study contributes to a better understanding of the gaucho market, as well as the opportunities and limitations in terms of consumer acceptance for businesses that wish to invest in this type of product. In addition, regarding the theoretical implications, the main one brought by this study is the growth of the bibliography regarding the consumers' attitude toward insect-based foods, in particular, from Rio Grande do Sul.

This study is limited by a qualitative analysis carried out with only 14 interviews, and by a quantitative analysis that while useful, is not exhaustive. A more sophisticated analysis (e.g., multinomial regression models) could provide further market implications on consumers' attitudes toward traditional and insect-based products. However, considering the novelty of the topic and scarce empirical studies in Latin America, our exploratory study provides novel and relevant information on consumers' attitudes toward food that can potentially contribute to dealing with an increasing demand for food.

Future studies can explore the attitude of consumers when more information is provided in terms of the products' benefits, both in relation to the environment and in relation to nutritional quality. Studies can investigate whether there is a relationship between green consumers and the acceptance of insect-based products. Besides, future studies should include representative samples, so that data can be generalized to the population.

## Data Availability Statement

The raw data supporting the conclusions of this article will be made available by the authors, without undue reservation.

## Author Contributions

JJ performed the analysis, MB and JJ conceived and designed the study. JJ, MB, and NC collected the data, contributed on analysis tools, interpreted, and wrote the manuscript. All authors contributed to the article and approved the submitted version.

## Conflict of Interest

The authors declare that the research was conducted in the absence of any commercial or financial relationships that could be construed as a potential conflict of interest.

## References

[1] FAO. Representante da FAO Brasil Apresenta Cenário da Demanda por Alimentos. Brasília: FAO (2017). Available online at: http://www.fao.org/brasil/noticias/detail-events/en/c/901168/ (accessed August 25, 2018).

[2] Kostecka J Koniecza K Cunha L. Evaluation of insect-based acceptance by representatives of polish consumers in the context of natural resources processing retardation. J Ecol Eng. (2017). 18:166–74. 10.12911/22998993/68301

[3] Yen AL. Edible insects: traditional knowledge or Western phobia? Entomol Res. (2009) 39:289–98. 10.1111/j.1748-5967.2009.00239.x

[4] Jongema Y. List of Edible Insect Species of the World. Wageningen: Laboratory of Entomology (2017). Available online at: https://www.wur.nl/en/Research-Results/Chair-groups/Plant-Sciences/Laboratory-of-Entomology/Edible-insects/Worldwide-species-list.htm (accessed June 1, 2021).

[5] Van Huis A Itterbeeck JV Klunder H Mertens E Halloran A Muir G. Edible Insects: Future Prospects for Food and Feed Security. FAO Forestry Paper No. 170. Rome: Food and Agriculture organization of the United Nations (FAO) (2013). p. 187. Available online at: http://www.fao.org/docrep/018/i3253e/i3253e.pdf (accessed September 07, 2018).

[6] Bukkens SGF. The nutricional value of edible insects. Ecol Food Nutr. (1997) 36:287–319. 10.1080/03670244.1997.9991521

[7] Defoliart GR. Insects as food: why the western attitude is important. Annu Rev Entomol. (1999) 44:21–50. 10.1146/annurev.ento.44.1.21 9990715

[8] Sheth J. Comportamento do consumidor. In: Czinkota MR, editor. Marketing as Melhores Práticas. São Paulo: Bookman (2001). p. 136–61.

[9] Schiffman L Kanuk L. Comportamento do Consumidor. 9. ed. Rio de Janeiro: LTC (2009).

[10] Sheth J Mittal B Newman B. Comportamento do Cliente – Indo Além do Comportamento do Consumidor. São Paulo: Atlas Publisher (2001).

[11] Mitshuhashi J. The future use of insects as human food. In: Durst PB Johnson DV Leslie RN Shono K. Forest Insects as Food: Human Bite Back. Bangkok: FAO of United Nations Regional Office for Asia and the Pacific (2010). p. 115–22.

[12] Pelchat ML Pliner P. “Try it, You'll like it”. Effects of information on willingness to try novel foods. Appetite. (1995) 24:153–16. 10.1016/S0195-6663(95)99373-8 7611749

[13] Bermúdez-Serrano IM. Challenges and opportunities for the development of an edible insect food industry in Latin America. J Insects Food Feed. (2020) 6:537–55. 10.3920/JIFF2020.0009

[14] Gomez-Luciano CA De Aguiar L Vriesekoop F Urbanc B. Consumers' willingness to purchase three alternatives to meat proteins in United Kingdom, Spain, the Dominican Republic and Brazil. Food Qual Prefer. (2019). 78:103732. 10.1016/j.foodqual.2019.103732

[15] Costa AIA Jongen WMF. New insights into consumer-led food product development. Trends Food Sci Technol. (2006). 17:457–65. 10.1016/j.tifs.2006.02.003

[16] Sarkar S Costa A. Dynamics of open innovation in the food industry. Trends Food Sci Technol. (2008) 19:574–80. 10.1016/j.tifs.2008.09.006

[17] Macfie H. Consumer-Led Food Product Development. Sawston: Woodhead Publishing (2007).

[18] Berenbaum MR. Bugs in the System. New York, NY: Basic Books (1995).

[19] Costa Neto EM. Insects as human food: an overview. Amazônica. (2013) 5:562–82. 10.18542/amazonica.v5i3.1564

[20] Mela DJ. Symposium on ‘Functionality of nutrients and behaviour’: food choice and intake: the human factor. Proc Nutr Soc. (1999). 58:513–21. 10.1017/S0029665199000683 10604182

[21] Flandrin J Montanari M. História da Alimentação. 7ª ed. São Paulo: Estação Liberdade Publisher (2013).

[22] Cheung T Moraes M. Inovação no setor de alimentos: insetos para consumo humano. Interações. (2016). 17:503–15. 10.20435/1984-042X-2016-V.17-N.3(12)

[23] Lucchese-Cheung T Aguiar LKD Da Silva RFF Pereira MW. Determinants of the intention to consume edible insects in Brazil. J Food Prod Market. (2020) 26:297–316. 10.1080/10454446.2020.1766626

[24] Pliner P Salvy SJ. Food neophobia in human. In: Shepherd R Raats M, editors. The *Psychology* of Food Choice. Oxfordshire: CABI Publishing (2006). p. 75–92.

[25] Rozin P. Cultural approaches to human preferences. In: Morley J Sterman B Walsh J, editors. Nutritional Modulation of Neural Function. Nova York: Academic Press (1998). p. 137–53.

[26] Creswell JW. Projeto de Pesquisa: Métodos Qualitativo, Quantitativo e Misto. Porto Alegre: Bookman (2007). p. 126.

[27] Austgulen MH Skuland SE Schjoll A Alfnes F. Consumer readiness to reduce meat consumption for the purpose of environmental sustainability: insights from Norway. Sustainability. (2018) 10:1–24. 10.3390/sul10093058

[28] Vasileiou K Barnett J Thorpe S Young T. Characterizing and justifying sample size sufficiency in interview-based studies: systematic analysis of qualitative health research over a 15-year period. BMC Med Res Methodol. (2018) 18:148. 10.1186/s12874-018-0594-7 30463515 PMC6249736

[29] Pliner P Hobden K. Development of a scale to measure the trait of food neophobia in humans. Appetite. (1992) 19:105–20. 10.1016/0195-6663(92)90014-W 1489209

[30] Kulma K Tumová V Fialová A Kourimská L. Insect consumption in the Czech Republic: what the eye does not see, the heart does not grieve over. J Insects Food Feed. (2020). 6:525–35. 10.3920/JIFF2020.0020

[31] Rumpold BA Langen N. Consumer acceptance of edible insects in an organic waste-based bioeconomy. Curr Opin Green Sustain Chem. (2020) 23:80–4. 10.1016/j.cogsc.2020.03.007

[32] Hartmann C Siegist M. Insect as food: perception and acceptance. Findings from currente research. Ernahrungs Umschau. (2017) 64:44–50. 10.4455/eu.2017.010

[33] Malhotra N. Pesquisa de Marketing: Uma Orientação Aplicada. 6. ed. Porto Alegre: Bookman Publisher (2012).

[34] Gorman B Primavera L. The complementary use of cluster and factor analysis methods. J Exp Educ. (1983). 51:165–68. 10.1080/00220973.1983.11011856

[35] House J. Consumer acceptance of insect-based foods in the Netherlands: academic and commercial implications. Appetite. (2016) 107:47–58. 10.1016/j.appet.2016.07.023 27444958

[36] Lensvelt E Steenbekkers L. Exploring consumer aceptance of entomophagy: a survey and experiment in Australia and Netherlands. Ecol Food Nutr. (2014). 53:543–61. 10.1080/03670244.2013.879865 25105864

[37] Lammers P Ulmann L Fiebelkorn F. Acceptance of insects as food in Germany: it is about sensation seeking, sustainability consciousness, or food disgust? Food Qual Prefer. (2019) 77:78–88. 10.1016/j.foodqual.2019.05.010

